# A new paradigm for musculoskeletal pain care: moving beyond structural impairments. Conclusion of a chiropractic and manual therapies thematic series

**DOI:** 10.1186/s12998-023-00484-2

**Published:** 2023-06-01

**Authors:** Julie M. Fritz, Alice Kongsted

**Affiliations:** 1 Department of Physical Therapy & Athletic Training and Associate Dean for Research, College of Health, University of Utah, Salt Lake City, UT USA; 2 Department of Sports Science and Clinical Biomechanics, University of Southern Denmark, Senior Researcher, Chiropractic Knowledge Hub, Odense, Denmark

**Keywords:** Musculoskeletal pain, Contextual factors, Self-management, Working alliance

## Abstract

This commentary closes the thematic series “A new paradigm for musculoskeletal pain care: moving beyond structural impairments”. The papers published in the series point to key aspects of shifting the paradigm of musculoskeletal care from clinician-led management often focused on addressing presumed structural anomalies to partnering with patients to find individual strategies that empower patients towards self-management. Several papers in the series highlighted the need for developing patient-centred models of care that respect individual patient’s needs and preferences. Also, the series pointed to different options for modes of delivery including mHealth and the challenges and opportunities they present for developing person-centred strategies. For health care to provide effective support for people with musculoskeletal pain conditions, there is a need to recognise that contextual factors, including a strong patient-provider alliance, clearly play an important, perhaps primary, role. Health care professions dealing with musculoskeletal pain conditions should engage in research to investigate effective ways to move this understanding into practice including how to train providers. We hope the work collected in this series will stimulate further questions and more research as musculoskeletal pain providers seek to make their care more person-centred.

## Background

It has long been recognised that musculoskeletal pain conditions are a result of a complex interaction of biological, psychological, and social factors that cannot be resolved by addressing structural injury or impairment alone. Rather, clinical guideline advocate a person-centred approach that supports patient self-management. Person-centred care necessitates consideration of the context within which care is provided, how patients interact with providers, and the cognitive and emotional responses of both patients and clinicians. These considerations have previously been dismissed as “non-specific” or part of a placebo effect, but increasing recognition of the clinical relevance of these contextual factors requires a re-conceptualization of their role [1]. Considering these factors is not in contrast to providing evidence-based treatments such as exercise therapy and manual therapies, but offers an opportunity to consider how clinician-led, evidence-based interventions may be integrated while supporting and encouraging patient self-management through person-centred care.

In July 2021, Chiropractic and Manual Therapy called for submissions to a thematic series under the heading “A new paradigm for musculoskeletal pain care: moving beyond structural impairments”. The intension was to contribute to discussions and reflections on ways that health care providers and systems that have traditionally focused on structural diagnosis and impairment can take a role in improving person-centred musculoskeletal pain care and facilitating self-management. With eight papers published in the series we will now discuss what we have learned from these contributions.

## Which topics were addressed?

The papers included in this thematic series use of variety of methodologies to examine important aspects of person-centred care that move beyond the paradigm of structural impairments (Table 1). Collectively the papers in this series identify and explore contextual factors that can impact outcomes irrespective of the specific structural interventions used including patients’ and providers’ beliefs, expectations and characteristics, the patient-provider working relationship, and characteristics of the therapeutic environment such as the mode of delivery (telehealth, mHealth, etc.). The topics addressed provide a reminder that contextual factors are not indescribable placebo effects but relate to all aspects of care than goes beyond addressing a specific structural mechanism.

**Table 1:** Summary of the paper included in the thematic series

Authors / Title	Methods	Aim	Authors’ Conclusion
Belton J, Birkinshaw H, Pincus T. Patient-centered consultations for persons with musculoskeletal conditions [4].	Personal account of a patient and narrative discussion	This article is structured in a way that reflects this collaboration [between the healthcare professional and patient], showcasing the importance of acknowledging lived experience, the sharing of information, and the dynamic interactions that shape a consultation, in combination with supporting scientific evidence.	This article has discussed several recommendations for clinicians to use in consultations for chronic pain… Ultimately, shared uncertainty between healthcare professionals and patients is the beginning of a journey together exploring the path of pain management. Accompanied by listening, validation, empathy and reassurance, a willingness to explore the paths together and accept the uncertainty of the journey is key to moving forward with chronic pain, for both healthcare professionals and people with pain.
Bronfort G, Maiers M, Schulz C, Leininger B, Westrom K, Angstman G, Evans R. Multidisciplinary integrative care versus chiropractic care for low back pain: a randomized clinical trial [8].	Randomized clinical trial	The purpose of this manuscript is to report the primary and secondary clinical outcomes of a randomized trial of mono-disciplinary chiropractic care, versus multidisciplinary integrative care for sub-acute and chronic low back pain.	Low back pain patients who received integrative care by a multidisciplinary integrative care team tended to have better outcomes than those who received chiropractic care. However, given the relatively small magnitude of between group differences and the extensive resources required to successfully manage and implement, the team based integrative care might not be worthwhile. More efficient models for addressing biopsychosocial care for low back pain should be explored with greater emphasis on addressing the full spectrum of related psychosocial mechanisms and ensuring equitable access for all.
Innes S, Goncalves G, Leboeuf-Yde C. Who are the chiropractic students favouring a limitless scope of practice? Exploring the relation-ship with personality, magical thinking, and academic achievement [9].	Cross-sectional survey of chiropractic students	The aim of our study was to explore whether there are any factors that help explain the adoption of views on chiropractic conservatism (ChiroCon) in an evidence-based chiropractic education environment and its association with an increased likelihood of using SMT for conditions without an evidence-base (e.g., non-MSK).	To prevent a mismatch between students and learning institutions we recommend that both those colleges that favor the old conservative approach of a limitless scope of practice or other types of magical approaches and institutions with a modern, MSK-only approach should screen for magical thinking to include or exclude potential students according to their requirements. Finally, the findings of this study explained less than 30% of the total variance, and this means that other factors are at play in determining clinical decisions that will require further investigation.
Ivanova D, Bishop FL, Newell D, Field J, Wals M. Mixed methods systematic review of the literature base exploring working alliance in the chiropractic profession [5].	Mixed methods systematic review	This mixed methods systematic review aimed to synthesise qualitative and quantitative evidence to study the nature and the role of working alliance within chiropractic consultations.	Strong working alliance (WA) requires ongoing negotiation of treatment goals and expectations alongside collaboration on a mutually agreed upon treatment plan. These processes of negotiation and collaboration are facilitated by, and may in turn strengthen, interpersonal bonds involving trust and mutual respect. Bordin’s formulation of WA has the potential to improve our understanding of chiropractor–patient relationships by providing a conceptual framework for thinking about the nature of the therapeutic relationship and how it can impact clinical outcomes through psychosocial pathways. Further primary research is needed to establish the nature, appropriate measurement, and consequences of WA in chiropractic care.
Joern L, Kongsted A, Thomassen L, Hartvigsen J, Ravn S. Pain cognitions and impact of low back pain after participation in a self-management program: a qualitative study [2].	Qualitative interviews with GLA:D® participants	The objective of this study was to gain insights into the possible shifts in the understanding of LBP and the sense of being able to manage pain among patients participating in the GLA:D® Back program.	Our results suggest that that the impact of having participated in the program relate to how the content of the program resonated with the individual patient’s experiences and prior understanding of LBP. Not all patients changed their understanding or came to internalise new under-standings during a 10-weeks program, however the results support existing evidence that an improved understanding of what LBP may translate into people being less negatively affected. Awareness of the ways individuals’ understanding of LBP interact with behaviour and physical activities appear central for providing adaptive professional support and meeting the individual needs.
Saragiotto BT, Sandal LF, Hartvigsen J. Can you be a manual therapist without using your hands? [6]	Commentary	This commentary discusses the use of telehealth by manual therapists.	Access to online information,. is everywhere and will continue to grow, making it the most powerful vehicle to spread information, including health information and potentially healthcare for individuals. We suggest that practitioners of manual medicine make telehealth part of their clinical toolbox where it makes sense and where there is evidence that it is beneficial for people who seek their care.
Sherriff B, Clark C, Killingback C, Newell D. Impact of contextual factors on patient outcomes following conservative low back pain treatment: systematic review [7].	Systematic review	This systematic review therefore aims to examine interventions which potentially modify known context factors (CFs) during conservative LBP care… to investigate their impact on patients’ pain intensity and physical functioning outcomes. Delineating the influence and role of CFs in usual care rehabilitation settings may assist in identifying which of these CFs demonstrates potential clinical utility and ethical approaches to rehabilitation.	In conclusion, this systematic review has demonstrated preliminary evidence to indicate explicitly leveraging CFs augments conservative LBP treatment. It identified CFs reducing pain intensity and improving physical functioning outcomes and extracted specific strategies with prospective clinical utility. The heterogeneity of interventions suggests modifying more than one CF may be more impactful. In essence, the practitioner’s therapeutic potency lies in their capacity to simultaneously provide physical, cognitive, and emotional care to influence the patient’s mindset and consequently their physiology.
Svendsen MJ, Nicholl BI, Mair FS, Wood K, Rasmussen CDN, Stochkendahl MJ. One size does not fit all: Participants’ experiences of the selfBACK app to support self-manage-ment of low back pain—a qualitative interview study [3].	Qualitative interviews with selfBACK participants	In this paper, we qualitatively explore the implementation of selfBACK on (i) factors influencing embedding, integrating, and sustaining engagement with the selfBACK app, and (ii) the self-perceived effects, acceptability, and satisfaction with the selfBACK app.	We identified a number of key factors involved in embedding, integrating and sustaining engagement with the selfBACK app. Participants were generally satisfied with the selfBACK app and many experienced positive effects. The high prevalence of LBP globally coupled with the advantages of providing help through an app offers opportunities to help countless people dealing with LBP in daily managing their pain. A range of factors should be considered to facilitate implementation of self-management of LBP or similar pain conditions. These findings should help inform development of future pain/LBP self-management apps.

Two papers in the series used qualitative methods to explore patients’ experience with interventions focused on promoting self-management of chronic back pain through a group-based education and exercise program [2] or an app-based education, exercise and physical activity interventions [3]. Belton and colleagues provide additional insights on the patient’s perspective of care by exploring the narrative of one patient and her experience with seeking care for persistent pain [4]. The critical nature of the patient-provider relationship, or working alliance, is further explored in the mixed methods systematic review by Ivanova and colleagues [5]. Advances in technology and changes in response to the COVID pandemic are accelerating the use of telehealth, which raises unique challenges for developing an effective patient-provider relationships. This and other important considerations for using telehealth delivery are explored in the commentary by Saragiotti and colleagues [6].

Issues addressed in this thematic series have important implications for clinical practice, as well as clinical research and the education of health care practitioners providing care for persons with musculoskeletal pain conditions. The systematic reviews by Ivanova and colleagues and Sherriff et al. [7] identify gaps in our knowledge on the impact of specific contextual factors such as working alliance, and expectations and beliefs about pain on outcomes. In addition, the review by Sherriff and colleagues highlights the need for rigorous studies examining the effectiveness of interventions attempting to modify contextual factors during conservative care. The randomized clinical trial from Bronfort et al. [8] included in this series provides one example comparing different models for delivering conservative care (unimodal versus integrated care) and including outcomes such as pain coping strategies and self-efficacy to examine outcomes related to the development of self-management skills in the participants. Finally, Innes and colleagues [9] examine the attitudes and beliefs of students in chiropractic education programs around the perceived mechanisms of structural interventions and professional identity.

## The main messages conveyed

The papers in this thematic series highlight several important issues for musculoskeletal pain care. First, contextual factors clearly play an important, perhaps primary, role in conservative care. Recognition of this reality is consistent with the biopsychosocial model and should promote further professional dialogue on how to provide care in a manner that optimizes patient-centred outcomes. The importance of contextual factors and the need to promote self-management do not obviate the role of interventions such as exercise or manual therapies. But our growing understanding of contextual factors should prompt us to consider how our traditional interventions may actually benefit patients. Beyond the structural, physiologic effects that exercise and manual therapies may have, we should consider their role in promoting patients’ self-efficacy, positive expectancies and stronger therapeutic bonds with providers. From this perspective the manner in which traditional interventions are provided may be more important than their underlying mechanical or physiologic rationale. For example, the way providers communicate with patients, set expectations and provide feedback may build the working alliance with patients and enhance their self-efficacy for self-management irrespective of the specific interventions used.

The papers in this series also make it clear that greater consideration of contextual factors that are common across interventions will make musculoskeletal pain care more personalized, not less. Accounting for patients’ needs for empathic, bi-directional communication and understanding of their individual pain experience creates the opportunity for providers to truly collaborate and keep their patients at the centre of their care and life. New delivery modes such as telehealth and mHealth strategies provide opportunities for innovative care models, but also raise unique challenges for developing working alliances and promoting person-centred care. The clinical skills necessary to form effective collaborative relationships with patients are not established, but it is clear that developing core competencies that optimize person-centred care will require different training strategies for clinicians and students at educational institutions.

Finally, the papers in this thematic series point to areas that researchers, clinicians, patients, and decision makers must collaborate around to improve the care provided to persons with musculoskeletal pain complaints. Emerging information calls attention to the critical nature of the patient-provider working alliance, yet there is presently little evidence on how to build and sustain alliances. Future research in this area must incorporate the perspectives of both patients and providers. The need for providers to facilitate and promote effective coping strategies and self-management skills for their patients is recognized, but optimal strategies for achieving these clinical objectives have not been identified. The challenge in shifting providers’ approach to patients from a perspective of fixing structural/mechanical anomalies to developing effective partnerships that help people engage in valued activities should not be under-estimated as it involves different skills as well as a shift in professional identity [10].

Also, the shift towards a more person-centred, comprehensive approach to musculoskeletal pain care offers opportunities for providers who are able to provide this care to play pivotal front-line roles. Ideally, care pathways would situate providers prepared to manage the complex biopsychosocial factors contributing to persistent musculoskeletal pain at the entry-point into health care systems as a strategy to promote self-management and reduce the risk for unwarranted escalation in care [11]. Implementing these care pathways will require collaboration with decision-makers and continuing professional development for providers. Along with evidence pointing to shared effect mechanisms across treatments for musculoskeletal pain conditions, [12, 13] the papers in this series thus point to the need to develop and test interventions to train clinicians to deliver person-centred care and ways to implement a shift in care pathways.

## The editors’ conclusions

The impact of chronic, persistent musculoskeletal pain on individuals and society, and the reality that interactions with health care systems too often exacerbate instead of alleviate concerns make it clear that a transformation away from clinician-led management towards management where persons with musculoskeletal pain play a key role in their own care is necssary [14]. The papers in this thematic series highlight key considerations to inform this necessary paradigm shift. The papers also point to several gaps in the evidence to inform the development of effective and sustainable health care for musculoskeletal pain. We hope the work collected in this series will stimulate further questions and more research as musculoskeletal pain providers seek to make their care more person-centred and educational institutions develop ways to facilitate person-centred care.

## Data Availability

Not applicable.
